# Supplementary material for: Loss of the centrosomal protein ALMS1 alters lipid metabolism and the regulation of extracellular matrix-related processes
Source: Biol Direct. 2023 Dec 8;18:84. doi: 10.1186/s13062-023-00441-2 (PMC10704752; doi:10.1186/s13062-023-00441-2)

Loss of the centrosomal protein ALMS1 alters lipid metabolism and the regulation of extracellular matrix-related processes

Brais Bea-Mascato[1,2], Eduardo Gómez-Castañeda[4], Yara E. Sánchez-Corrales[3], Sergi Castellano[3,5], Diana Valverde[*1,2]

[1] CINBIO, Universidad de Vigo, 36310 Vigo, Spain.

[2] Grupo de Investigación en Enfermedades Raras y Medicina Pediátrica, Instituto de Investigación Sanitaria Galicia Sur (IIS Galicia Sur), SERGAS-UVIGO, Vigo, Spain

[3] Genetics and Genomic Medicine Department, Great Ormond Street Institute of Child Health, University College London, London, United Kingdom

[4] Molecular and Cellular Immunology Section, Great Ormond Street Institute of Child Health, University College London, London, United Kingdom

[5] UCL Genomics, Zayed Centre for Research into Rare Disease in Children, University College London, London, United Kingdom

[*]Correspondence: Diana Valverde. Email dianaval@uvigo.es

CINBIO Facultad de Biología, Universidad de Vigo, Campus As Lagoas-Marcosende s/n, 36310 Vigo, Spain

Tel +34 986 811 953

Supplementary Figures:

A

Sample Name	% Assigned	M Assigned	% Aligned	M Aligned	% Dups	% GC	Length	M Seqs
C1	15.6%	11.6	47.6%	12.5	66.8%	52%	122 bp	26.3
C2	20.1%	14.2	51.1%	15.3	70.8%	53%	140 bp	29.8
C3	19.0%	10.8	48.9%	11.6	63.6%	51%	100 bp	23.6
KO1	16.1%	19.0	46.0%	20.4	62.3%	54%	111 bp	44.4
KO2	20.6%	11.5	53.5%	12.3	52.0%	52%	95 bp	23.1
KO3	24.5%	16.1	56.8%	17.2	63.1%	53%	125 bp	30.3

B

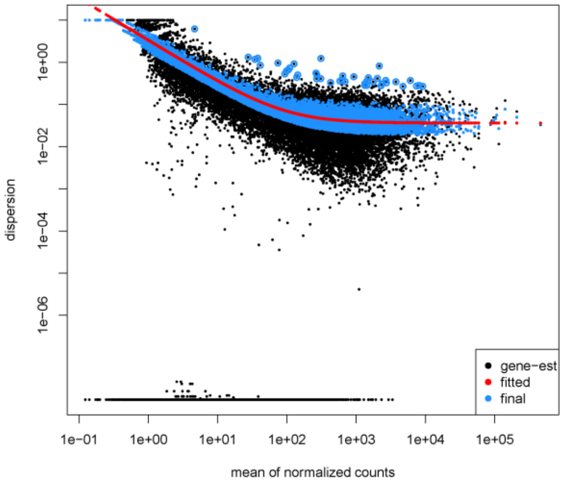

C

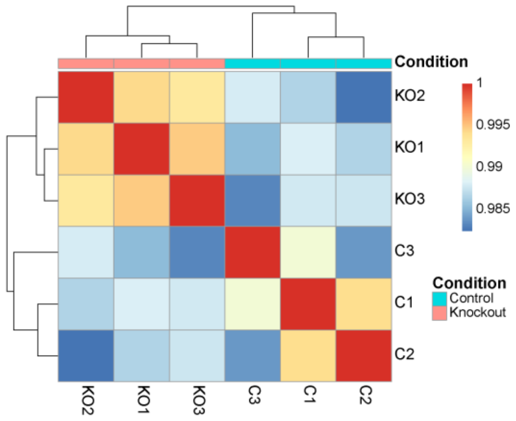

D

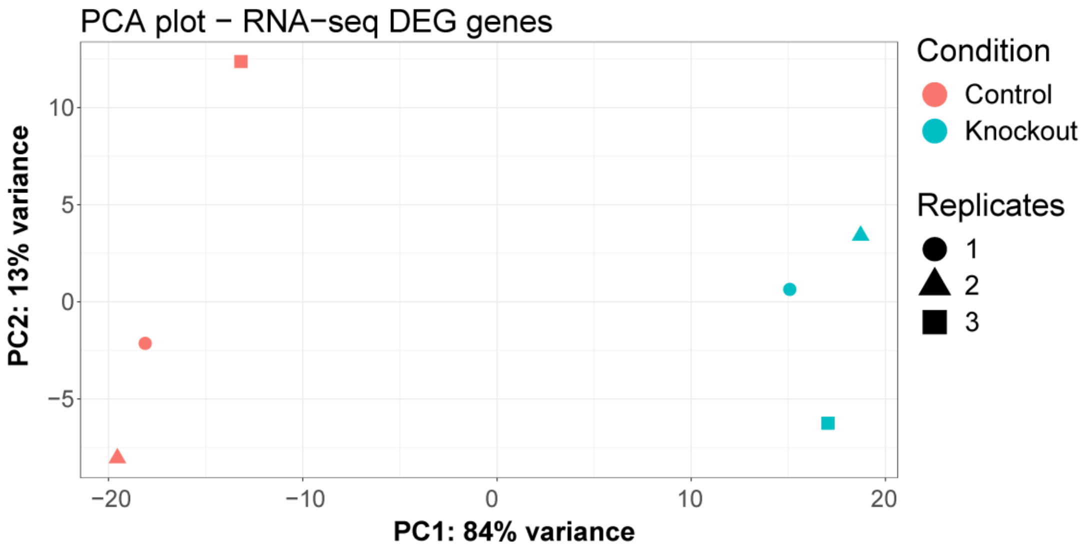

Supplementary Figure S1. RNA-seq quality controls. **(A)** Table with the quality controls, showing percentage of assigned, aligned and duplicated reads, GC percentage and average read length. **(B)** Dispersion plot showing the decrease in variance as the normalised mean number of counts in each gene increases. **(C)** Correlation matrix showing similarity between biological replicates of each genotype. **(D)** PCA plot of the RNA-seq samples, where PC1 (genotype) accounts for 84% of the variance between samples.

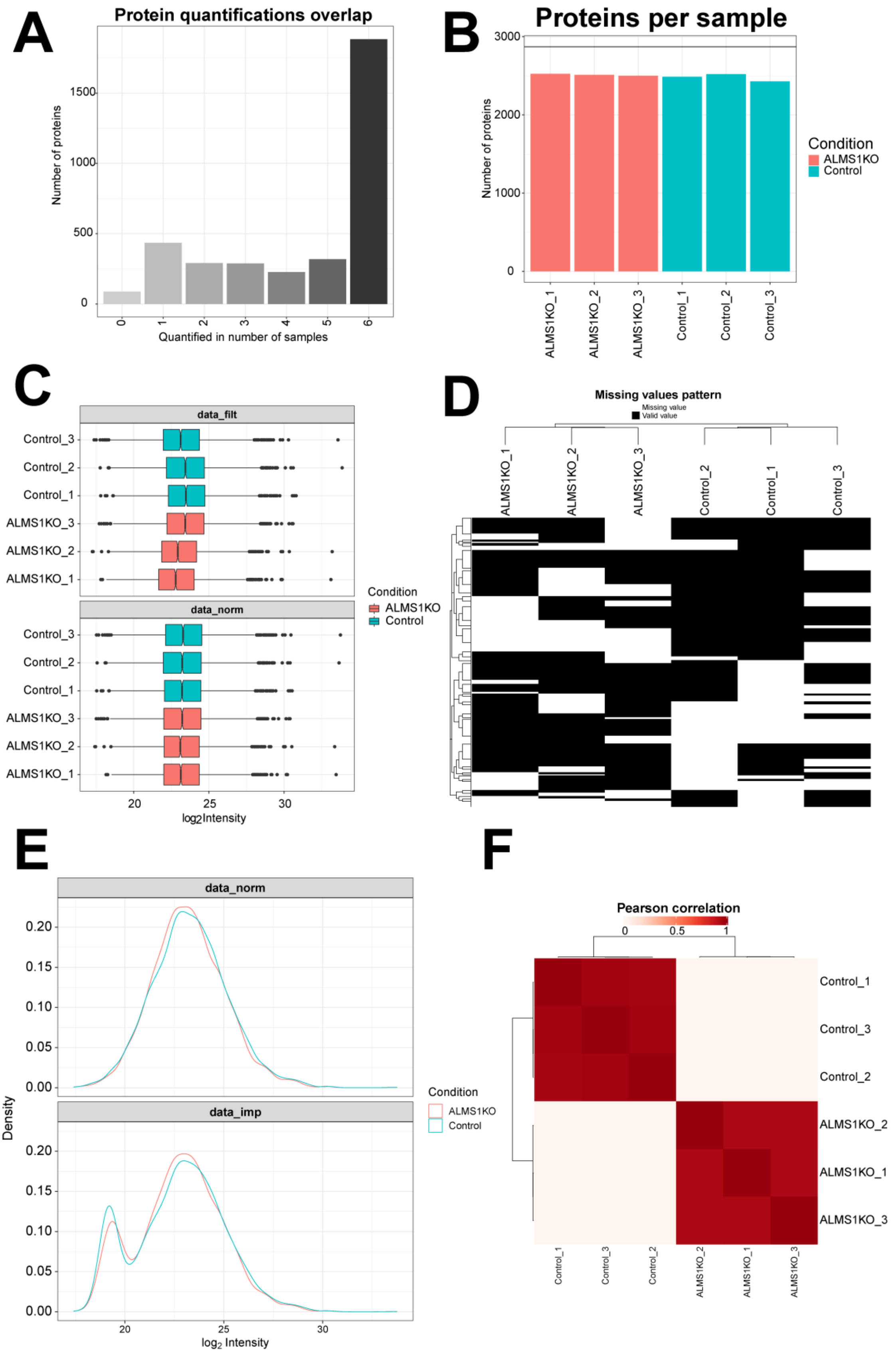

Supplementary Figure S2. Proteomics quality controls **(A)** Overlap of proteins quantified in each sample. A total of 1,800 proteins were quantified in all samples. **(B)** Number of proteins

identified in each sample. Approximately 2,870 proteins were identified in each sample. **(C)** Box-plots showing the distribution of log2 Intensity of each protein per sample before and after applying VSN normalisation. **(D)** Heatmap showing the pattern of non-random missing values prior to imputation. **(E)** Density-plot of the distribution of the average log2 values of the intensities between WT and KO before and after applying the Minprob imputation with an FDR > 0.01. **(F)** Correlation matrix showing similarity between biological replicates of each genotype.

Original Western Blots:

p-AKT:

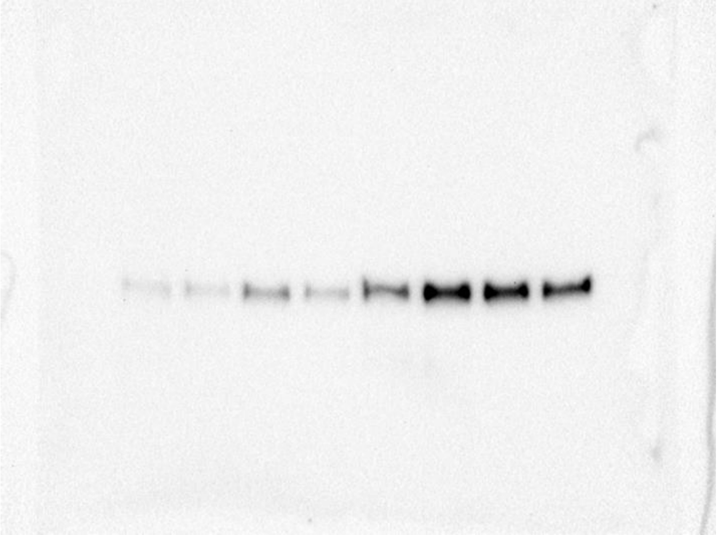

AKT:

The first well was a bad load of WT time 0.

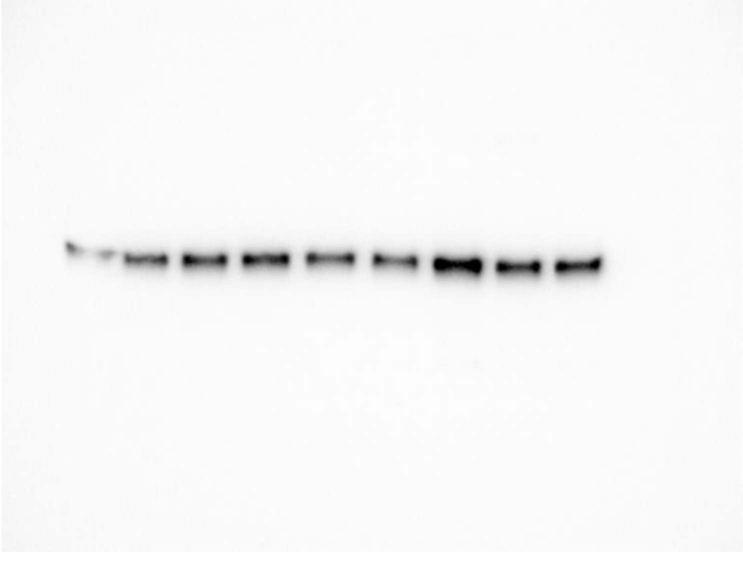

Supplement: Supplementary file 1 — Supplementary Material 1 [file 13062_2023_441_MOESM1_ESM.pdf]
